# Magnetic methods in studies of new superconducting hydrides in a diamond anvil cell

**DOI:** 10.1093/nsr/nwae005

**Published:** 2024-01-05

**Authors:** Viktor V Struzhkin, Ho-kwang Mao

**Affiliations:** Center for High Pressure Science & Technology Advanced Research, China; Center for High Pressure Science & Technology Advanced Research, China

## Abstract

This short perspective article summarizes the growing experimental evidence supporting the original claims about hydrogen-rich “superhydrydes” as members of a new family of nearly room temperature BCS superconductors, with hydrogen sub-lattice pre-compressed to the metallic and superconducting state, exactly as predicted in earlier and more recent theoretical works.

In recent years, we have witnessed great success in discoveries of high-transition-temperature (*T_c_*) hydrides using high-pressure experiments. This has been achieved by creating metallic hydrogen ‘alloys’ with other elements across the periodic table. Although the original idea suggested by Neil Ashcroft [1] was to use alloying to reduce the pressure required for producing pure metallic hydrogen, the pressures required to stabilize these hydrides are still so high that *in situ* measurements of the complete expulsion of the magnetic field (the Meissner state)—a defining character of superconductivity—are difficult to obtain. Such a deficit in the Meissner effect studies has provoked multiple critiques of claims of superconductivity in these new hydride materials and stimulated new technical developments to meet these challenges. In this perspective article, we will provide a short summary of the results obtained with four alternative techniques that were used to probe the magnetic field expulsion and the Meissner state in a diamond anvil cell (DAC), which is the only capable high-pressure vessel for studying the high-*T_c_* superconducting hydrides at very high pressures.

Figure 1 shows an overview of the magnetic techniques that were used to probe the magnetic field expulsion and the Meissner state in a DAC in studies of several superconducting superhydrides.

**Figure 1.**
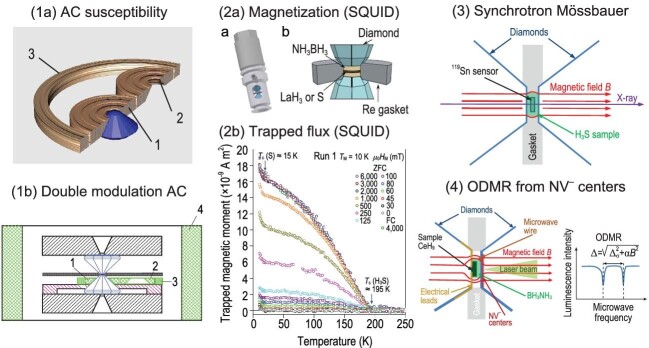
(1a) The standard AC susceptibility technique uses the pick-up coil (1), the compensation coil (2) and the excitation coil (3) to measure the expulsion of the magnetic field from the sample volume (the Meissner effect) [2]. (1b) The double-modulation method uses an additional external coil (4) which is operating at another (lower) frequency to reduce the background signal from the sample environment [3]. (2a) The set-up for the magnetization measurements includes the special non-magnetic DAC designed to fit a bore in a magnet belonging to a SQUID magnetometer—see Ref. [4]. (2b) The set-up for the trapped flux measurements is similar to (2a). The temperature dependence of the trapped flux is reproduced from Ref. [5] (FC: field cooling; ZFC: zero field cooling). (3) The schematic of the syncrotron Mössbauer experiment on the magnetic field expulsion from the tin sensor embedded in the H_3_S sample (reproduced from Ref. [6]). (4) The summary of nitrogen-vacancy (NV^−^) magnetometry details in a DAC—Ref. [7]. An example of the ODMR frequency scan is also shown illustrating the dependence of the ODMR splitting on stress (Δ_σ_) and magnetic field (B). (2a) and (2b) are reproduced from the referenced work under the terms of the Creative Commons CC BY license (https://creativecommons.org/licenses/), which permits unrestricted use, distribution and reproduction in any medium, provided the original work is properly cited.

A brief explanation of the four methods from Fig. 1 is given below.

(1a) The alternating current (AC) susceptibility method was used to measure magnetic susceptibility signals from the sulfur hydride sample showing superconductivity at T_c_ = 183 K and at high pressures of ∼150 GPa [2].
(1b) The AC susceptibility studies using the double-modulation technique were reported for LaH_10_ by our group [3] and also in a recent arxiv publication for the carbonaceous sulfur hydride (C–S–H) system by Pasan *et al.* [8]. It should be noted that the signal-to-noise ratios in these measurements are too low for performing systematic studies of superhydride superconductors prepared by using the laser heating technique. The reason for this is the failure in preparing a bulk superconducting sample of the required size (see for example the recent results on homogeneity of the laser-heated samples as obtained by using the nitrogen-vacancy (NV^−^) technique [7]). Future developments of the double-modulation technique at higher operating frequencies could improve the signal-to-noise ratio and may allow studies of samples of smaller spatial dimensions.
(2a) The magnetic moment in sulfur hydride at the record-breaking critical temperature *T_c_* [9] was directly measured in the non-magnetic DAC using the superconducting quantum interference device (SQUID) magnetometer; the measurements were performed in the Magnetic Properties Measurements System from Quantum Design. A subsequent paper by Minkov *et al.* addressed the measurements of the penetration of the magnetic field in the H_3_S, and in the LaH_10_ samples [4].
(2b) The aforementioned SQUID technique was also used in the trapped flux measurements in H_3_S and in LaH_10_ [5], which provided additional information about the superconducting state and was applied to study the superconductivity beyond the Meissner state. The authors [5] noted that the measurements of the magnetic moment from the trapped flux do not have a contribution from the background signal since, in such measurements, the external magnetic field is zero. The different regimes of the measurements of the trapped flux provide unambiguous proof of the superconducting states in H_3_S and LaH_10_ [5].
(3) Troyan *et al.* [6] applied the nuclear forward scattering (syncrotron Mössbauer) technique using the tin sensor embedded in the H_3_S sample to detect the expulsion of the magnetic field from the sample. The magnetic field used by such a technique is of the order of 0.65 T, which is too high for detecting the Meissner state because typical penetration fields in disk-shaped samples are <0.4 T, as reported by Minkov *et al.* [4]. The data reported by Troyan *et al.* [6] show clear expulsion of the magnetic field from the superconductor volume at lower temperatures when the penetration fields and the lower critical field *H_c1_* become comparable to the probing magnetic field of 0.65 T [6]. It is conceivable that, by optimizing the sample geometry and the demagnetizing factor, the penetration field could be brought closer to the *H_c1_* value and the Meissner state would be accessible in such future experiments.
(4) A relatively new method for detecting the expulsion of the magnetic field under pressure is based on the sensitivity of the NV^−^ centers in a diamond to the local magnetic field (NV^−^ centers embedded in a diamond anvil represent an example of a proximity sensor). This method was recently used for the studies of the CeH_9_ superhydride up to ∼140 GPa [7]. The method has very high spatial resolution due to the fact that the sensitive area is limited only by the size of the focused laser beam that is used for probing the optically detected magnetic resonance (ODMR). Looking forward, this work may be extended in a number of directions. It would be interesting to revisit other high-pressure superconductors, such as LaH_10_, H_3_S and other superhydrides, for which prior magnetic measurements have been limited mostly to the non-local probes described above (SQUID and AC susceptibility).

The growing evidence of the magnetic field expulsion and the Meissner effect in superhydrides is slowed down by very small signal levels to be extracted from high background as well as noise. However, existing publications are already sufficient to demonstrate the Meissner properties and also to extract important parameters such as the lower critical field *H_c1_* of superhydride superconductors. The evidence for these new superconducting superhydrides was extensively criticized by Prof. Jorge Hirsch—see for example Ref. [10] and the references within it. The critique is helpful for improving old techniques and for providing incentive to novel approaches to probing the Meissner state at very high pressures. A point-to-point answer to all Hirsch's criticisms would be useful but will have to wait; it will take at least 10 times longer to present that discussion and is inconsistent with the goal of the present paper, which aims to give novices and experts a concise perspective on high-pressure magnetic measurements regarding superconductivity. In our opinion, it has become evident that growing experimental evidence supports the original claims about superhydrides as members of the new family of nearly room-temperature Bardeen-Cooper-Shrieffer superconductors, with a hydrogen sub-lattice pre-compressed to the metallic and superconducting state, exactly as predicted in earlier and more recent theoretical studies.
